# Efficacy and Safety of Finerenone in Kidney Transplant Patients

**DOI:** 10.3390/jcm14238296

**Published:** 2025-11-22

**Authors:** Serdar Kahvecioglu, Huseyin Celik, Asena Serap Yalcinkaya, Yavuz Ayar, Nimet Aktas, Ozger Akarsu

**Affiliations:** 1 Nephrology Department, Bursa Yüksek İhtisas Trainig and Research Hospital, Bursa 16310, Turkey; serapp.yalcinkaya@gmail.com (A.S.Y.); yavuzayar@hotmail.com (Y.A.); nimetaktas@gmail.com (N.A.); ozgerakarsu@yahoo.com (O.A.); 2 Nephrology Department, Bursa Acıbadem Hospital, Bursa 16210, Turkey; huseyin_c66@yahoo.com.tr

**Keywords:** finerenone, kidney transplantation, mineralocorticoid receptor antagonists, proteinuria, renal function

## Abstract

**Background:** Finerenone has emerged as a promising nonsteroidal mineralocorticoid receptor antagonist for patients with chronic kidney disease (CKD), yet its safety and efficacy in kidney transplant recipients remain unstudied. **Methods:** A total of 1750 kidney transplant recipients were screened, and 39 were prescribed finerenone alongside angiotensin-converting enzyme inhibitors or angiotensin II receptor blockers. Fifteen patients who met inclusion criteria and used finerenone consistently for at least six months were compared with 15 matched controls. Proteinuria, renal function, serum potassium, and other laboratory and clinical parameters were assessed at baseline and at 1, 3, and 6 months. **Results:** Finerenone was discontinued in two patients (5.1%) due to flushing and headache. Severe hyperkalemia occurred in four patients (10.2%). In the finerenone group, proteinuria significantly decreased at all time points (*p* < 0.05), with a 40% reduction at six months. No significant changes in estimated glomerular filtration rate or serum creatinine were observed. **Conclusions:** Finerenone is a promising adjunct therapy in kidney transplant patients for reducing proteinuria without impairing renal function for those patients who can tolerate it. However, in the early phase of treatment initiation, patients should be closely monitored for adverse effects including hyperkalemia.

## 1. Introduction

Kidney transplantation is undoubtedly the first treatment option that should be preferred among renal replacement therapies [1]. Although the general condition of patients after kidney transplantation is satisfactory, renal functions may deteriorate due to early and late complications. Many factors such as ischemia reperfusion injury, oxidative stress, toxicity due to drugs used, and recurrence of the primary disease may cause this condition [2]. This deterioration in renal function typically manifests as elevated creatinine levels and proteinuria. Currently, there is no definitive approach to prevent the progression of chronic changes detected by biopsy. In recent years, in patients with chronic kidney disease (CKD) without transplantation, promising results have been achieved with the addition of sodium-glucose co-transporter 2 inhibitors (SGLT2-Is), glucagon-like peptide-1 (GLP-1) agonists, and new-generation nonsteroidal mineralocorticoid receptor antagonists (MRAs) such as finerenone, or combinations of these drugs, in addition with angiotensin-converting enzyme inhibitors (ACEIs) or angiotensin II receptor blockers (ARBs) [3,4,5,6]. However, strong evidence on the efficacy and safety of these agents in transplant recipients is required because kidney transplant patients have generally been excluded from major studies. Despite this, favorable results in CKD patients, animal studies, and small-scale clinical trials and case reports suggest that these drugs can also be used in kidney transplant patients [7,8,9,10,11].

Many studies have shown that mineralocorticoid receptors (MRs) become overactive in cardiac and renal diseases [12]. As a result, it has been observed that glomerular and tubular damage in the kidney increases, renal blood flow decreases, and proteinuria increases [13,14]. However, there are also studies showing that vascular and cardiac damage may increase. It has been shown that the use of MRA can be beneficial in blocking this activation [15]. The new generation nonsteroidal MRA finerenone, which binds to MR more effectively and selectively, has no central nervous system penetration and sexual side effects, has very little effect on blood pressure, and has been shown to provide significant benefits in chronic kidney disease [5]. It is thought that its rate of causing hyperkalemia, which is the most feared issue in this group of patients, may be at more acceptable levels compared to other drugs containing MRAs [16,17].

Based on the hypothesis that finerenone could provide beneficial effects such as a reduction in inflammation, fibrosis, oxidative stress, proteinuria, and blood pressure in transplant recipients, we aimed in this study to investigate the efficacy and safety of finerenone in kidney transplant patients.

## 2. Materials and Methods

### 2.1. Participants

Our study was conducted by screening a database of 1750 kidney transplant patients from two centers. All patients, regardless of the etiology of the primary kidney disease, both diabetic and non-diabetic, were evaluated.

#### 2.1.1. Inclusion Criteria

Age between 18–65 years.

Having a kidney transplant at least 1 year ago.

Estimated glomerular filtration rate (eGFR) > 25 mL/min.

Receiving ACEI or ARB therapy.

Protein creatinine ratio in spot urine (PCR) > 300 mg/g.

Additionally, patients who had been using 10–20 mg/day active finerenone for at least 6 months and whose baseline serum potassium was not above 5 mg/dL were included in the patient group.

#### 2.1.2. Exclusion Criteria

Rejection episode within the last 3 months.

Surgical intervention within the last 3 months.

Changes in immunosuppressive therapy within the last 3 months.

Receiving albumin infusion.

Unstable clinical condition.

Using potassium-sparing diuretics, potassium supplements, or potassium-lowering treatments.

Active infections.

### 2.2. Study Design

In this study, which was designed retrospectively, out of the 1750 screened patients, 39 were identified to have been prescribed finerenone at a dose of 10 mg/day.

**Figure 1:**
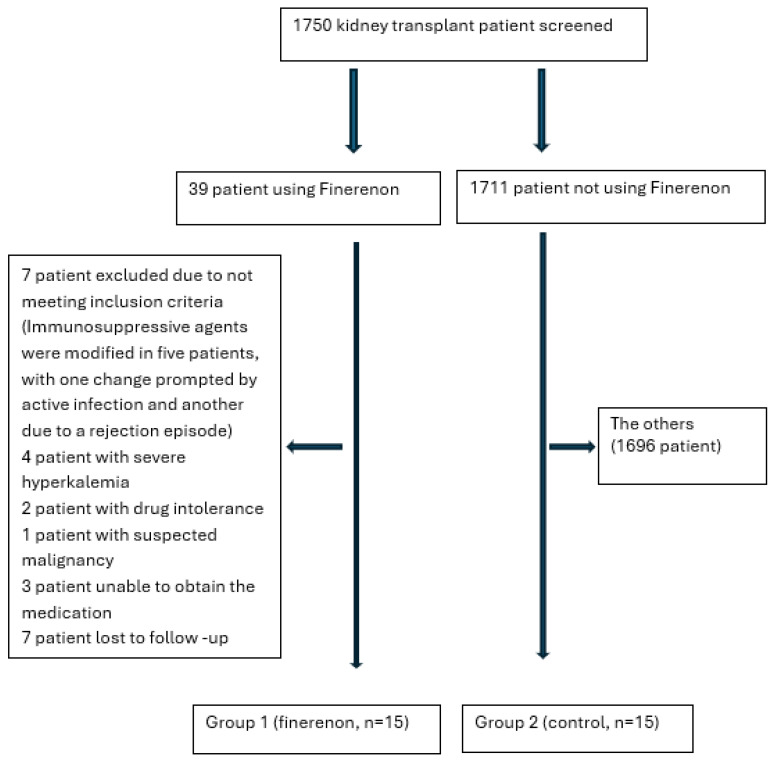
Materials and methods flowchart.

During follow-up, 15 patients were included in the finerenone group (GROUP 1). Among these, the dose was increased to 20 mg in 10 patients who tolerated the treatment well (according to serum potassium level). An equal number of matched patients meeting inclusion/exclusion criteria and with similar demographic and laboratory parameters were included as the control group (GROUP 2) (Figure 1). A one-to-one matching technique was applied for the pairing of patients and controls according to age, sex, eGFR, and proteinuria levels.

### 2.3. Data Collection and Follow-Up

The baseline demographic data of the patients in GROUP 1 who used finerenone and were included in the study, such as kidney transplantation type, presence of diabetes, serum urea, serum creatinine, serum potassium, eGFR, uric acid, alanine amino transferase, and the medications they used, were recorded from the routine visit information on the day treatment with finerenone was started. The data from the visits of the patients who used their medication regularly, which were closest to the 1st, 3rd, and 6th months, were also taken for evaluation. The patients in GROUP 2, who were included as the control group, were selected if their clinical conditions were stable and their proteinuria, eGFR, potassium levels, and demographic characteristics were compatible. The first value of these patients was accepted as the starting point in order to fit the averages of the patient group. The laboratory and demographic data from the closest follow-up visits at the 1st, 3rd, and 6th months were recorded. eGFR values were calculated according to the Chronic Kidney Disease Epidemiology Collaboration (CKD-EPI) formula, and proteinuria was calculated according to the protein/creatinine ratio in spot urine. Blood pressure assessment was not performed due to the lack of standardization, including differences in measurement devices, variability among personnel conducting the measurements, and incomplete documentation in patient records.

### 2.4. Ethics Statement

The study was approved by a local ethics committee on 26.03.2025 (decision number 2024-TBEK 2025/03-05). The study adhered to the Declaration of Helsinki and Good Clinical Practice guidelines.

### 2.5. Data Analysis

Data were collected from electronic medical records. Normality of continuous variables was tested using the Shapiro–Wilk test. Descriptive statistics were presented as percentages for categorical variables and as median, minimum–maximum, and 25th–75th percentiles for continuous variables.

For repeated measures within dependent groups, the Friedman test was used. If the Friedman test indicated statistical significance, post hoc pairwise comparisons were performed using the Wilcoxon signed-rank test. Categorical variables between groups were compared using the Chi-square test. Bonferroni correction was applied for multiple comparisons.

All statistical analyses were performed using IBM SPSS Statistics version 26.0 (IBM Corp., Armonk, NY, USA). A type I error rate of 5% (*p* < 0.05) was considered statistically significant.

## 3. Results

In the study, data were obtained from 15 patients who met the inclusion and exclusion criteria and used finerenone and 15 control patients with compatible demographic and laboratory data from 1750 kidney transplant patients (Figure 1). In the finerenone group, the median age was 42 (24–61) years, the transplantation period was 58 (13–150) months, and 33% of the patients were female, while in the control group, the mean age was 45 (19–59) years, the transplantation period was 80 (12–142) months, and 60% of the patients were female (*p* > 0.05). In the finerenone group, 1 patient and in the control group, 2 patients were using cyclosporine + mycophenolate mofetil + low-dose steroids, and all other patients were using tacrolimus + mycophenolate mofetil + low-dose steroids. There was no statistically significant difference in baseline values between the groups (*p* > 0.05) (Table 1).

**Figure 2:**
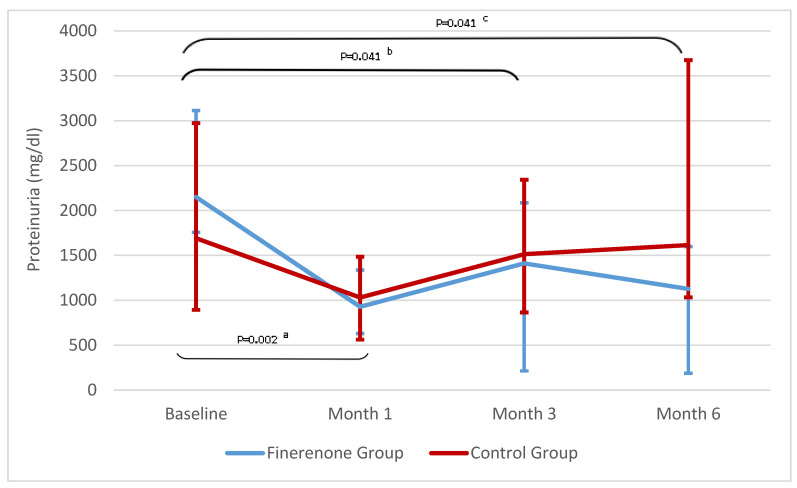
**Changes in proteinuria over time.** Data are presented as median (25p–75p). ^a^ Baseline–month 1 in GROUP 1, ^b^ Baseline–month 3 in GROUP 1, ^c^ Baseline–month 6 in GROUP 1.

A statistically significant decrease in proteinuria was found in the patients using finerenone; this was more pronounced in the 1st month (*p* < 0.002) throughout the follow-up period (*p* < 0.05). This decrease in proteinuria in the finerenone group was at the level of 40% at the end of the 6-month follow-up. There was no significant difference compared to the control group (Figure 2, Table 2).

The Friedman test showed a significant difference among the three time points in GROUP 1 (χ^2^ = 13.588, *p* = 0.004). Post hoc Wilcoxon signed-rank tests revealed significant differences between baseline and the first month (*p* = 0.002), between baseline and the third month (*p* = 0.041), and between baseline and the sixth month (*p* = 0.041), but not between the third and sixth months (*p* = 0.43), after Bonferroni correction.

**Figure 3:**
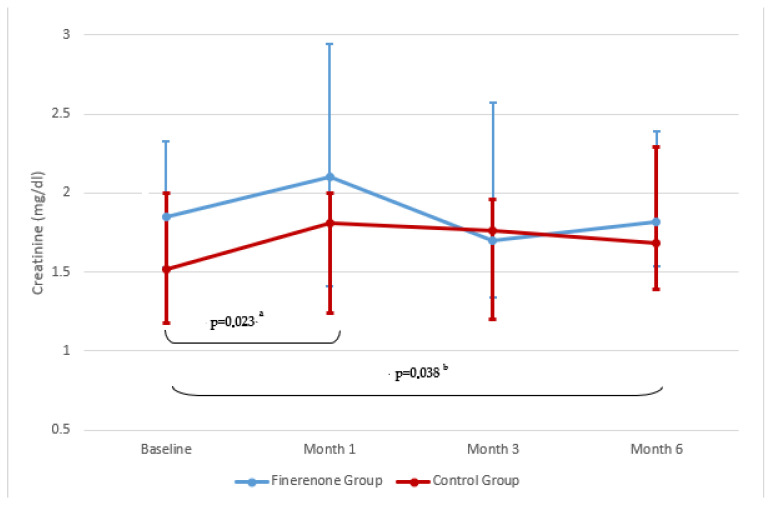
Changes in serum creatinine over time. Data are presented as median (25p–75p). ^a^ Baseline–month 1 in GROUP 2, ^b^ Baseline–month 6 in GROUP 2, creatinine levels showed a statistically significant decrease from baseline to 1 month (Z = −2.273, *p* = 0.023) and from baseline to 6 months (Z = −2.074, *p* = 0.038).

**Figure 4:**
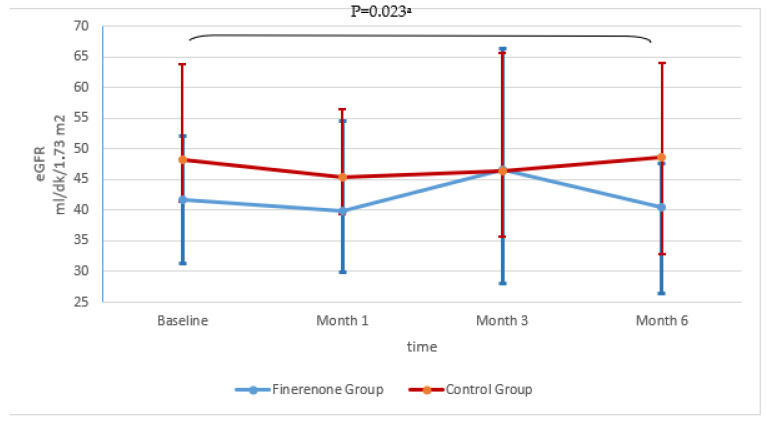
Changes in serum estimated glomerular filtration rate (eGFR) over time. Data are presented as median (25p–75p). ^a^ Baseline–month 6 in GROUP 2, eGFR levels showed a statistically significant decrease from baseline to 6 months (Z = −2.272, *p* = 0.023).

In terms of renal functions, it was observed that creatinine levels increased slightly in the first month in the finerenone group (*p* = 0.055) but decreased to near-baseline levels in the 3rd and 6th months. The change in eGFR was not statistically significant and remained stable. In the control group, there was a significant creatinine increase compared to the baseline in the 1st and 6th months. Statistical significance was found in eGFR compared to the baseline in the 6th month (*p* = 0.023). No significant difference was found in the comparison between the groups (*p* > 0.05) (Figure 3 and Figure 4, Table 2).

**Figure 5:**
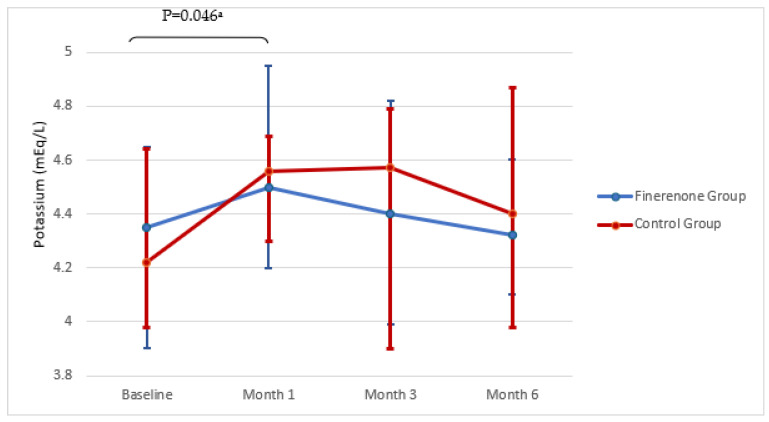
Changes in serum potassium over time. Data are presented as median (25p–75p). ^a^ Baseline–month 1 in GROUP 1. In GROUP 1, potassium levels showed a statistically significant increase from baseline to 1 month (Z = −1.992, *p* = 0.046).

When all patients who were started on finerenone were considered, the drug was discontinued due to side effects (flashing and headache) in 2 out of 39 patients (5.1%). One of the most curious issues when using MRA, significant hyperkalemia (>6 mEq/L) occurred in 4 (10.2) patients, and the drug was discontinued. During the treatment, it was observed that 3 out of 15 patients in the finerenone group with a baseline value of ≤5 mEq/L had asymptomatic mild potassium elevation in the 1st month (maximum 5.7 mEq/L), and 3 patients in the 3rd month (maximum 5.5 mEq/L). The potassium increase in the 1st month was significant, and there was a decrease toward the initial levels in the 3rd and 6th months (≤5 mEq/L). There were no patients with hyperkalemia leading to death, hospitalization, or dialysis. When the potassium changes in the 1st, 3rd, and 6th months of the study were compared with the control group, it was observed that there was no statistical difference (*p* > 0.05) (Figure 5, Table 2).

We did not find any statistical difference in the use of finerenone in patients using tacrolimus in terms of drug level in the comparison between groups or within the groups (*p* > 0.05). The comparisons of other laboratory parameters are shown in Table 2.

## 4. Discussion

This study is the first to investigate the potential efficacy of finerenone in kidney transplant recipients.

The presence of proteinuria is one of the main indicators of kidney damage in kidney transplant recipients, as in kidney diseases [18,19]. Studies have shown that proteinuria can be observed in up to 40% of kidney transplant recipients [20]. Many guidelines state that the use of SGLT2I, GLP1 agonist, or finerenone, or their combination therapies in addition to ARBs, especially in chronic kidney disease patients with heart failure or diabetes, are beneficial and should be added to treatment algorithms [4,21,22]. However, since kidney transplant patients were considered as an exclusion criterion in all large studies conducted with drugs known to be effective in renal protection, we did not have studies to guide us [5]. In our evaluation of the 15 patients who used finerenone and had regular follow-ups among the 1750 kidney transplant patients that we followed, and the same number of control group patients who did not have any statistical differences between them, we found that the use of finerenone provided a 40% decrease in proteinuria at the end of the 6-month follow-up and that this decrease was statistically significant compared to the baseline throughout the follow-up period, especially in the first month. In a meta-analysis evaluating 19 studies, it was observed that finerenone added to renin-angiotensin-aldosterone system blockers in non-transplant chronic kidney disease patients provided a 38.7% decrease in proteinuria, and the fact that almost the same result was obtained in our study suggests that the drug may be effective in kidney transplant patients as in non-transplant CKD [8,23]. As in the FIGARO-DKD study, it is observed that the effectiveness of the drug increases as the amount of proteinuria increases [5,23,24]. In a retrospective study conducted with spironolactone, a nonselective MRA, in kidney transplant recipients, it was detected that a statistically significant decrease in proteinuria was achieved after 6 months of treatment in patients with proteinuria over 1 g [8]. The fact that the average proteinuria was over 2 g/day in our study supports these studies and may have contributed to our results being more significant.

When we look at eGFR, which is one of the main indicators in the evaluation of renal functions, a slight decrease in eGFR was observed in the finerenone group as of the first month after starting the drug, and it returned to baseline values by the end of the sixth month. Serum creatinine also has a course parallel to eGFR, and the significant creatinine increase detected in the control group at the end of the six-month follow-up was not seen in the group receiving the drug. This situation observed in our kidney transplant patients is similar to the findings in studies conducted in other CKD groups without kidney transplantation. There are studies showing that MRAs are effective, especially in patients with eGFR values >30 mL/min/1.73 m^2^, and that this effect decreases as eGFR decreases [25,26,27,28]. When the data of the FIDELITY study, conducted with finerenone and examining over 13,000 CKD stage 1–4 patients, were evaluated, it was determined that there was a significant benefit in renal outcomes, including GFR, in the group receiving the drug [8]. In the recently concluded SPIREN study, which investigated renal function, proteinuria, and renal allotransplant fibrosis with the addition of spironolactone, a nonselective MRA, to standard treatment in kidney transplant recipients, with a follow-up of 3 years, no significant benefit was found [29]. However, there is no study conducted with selective MRAs. In our study, it was observed that the use of finerenone in kidney transplant patients, as in other chronic kidney disease patients, did not have at least a negative effect on eGFR, and the significant creatinine increase seen in the control group was not observed in the group receiving the drug.

The most feared condition in all studies conducted with MRAs is hyperkalemia. ACEIs and ARBs are generally used intensively in patients with impaired renal function or proteinuria. This situation further increases the risk of hyperkalemia. It has been shown that MRAs have a greater risk of hyperkalemia, especially in patients with high proteinuria, low GFR, and high baseline potassium levels [30,31]. Several studies have shown that finerenone causes significantly less hyperkalemia compared with spironolactone [32,33]. The main reasons for this may be the short half-life of the drug, the absence of active metabolites, and the mechanism of action of MR, which includes different receptor blockades and gene expression in organs such as the kidney and heart [34]. Finerenone is also superior to spironolactone in terms of gynecomastia [29,35]. In our study, severe hyperkalemia occurred in 10.4% of patients starting finerenone. Compared to the FIDELITY (1.7%) and FINE-REAL (5%) studies, our study demonstrated a higher risk of hyperkalemia [24,36]. However, mild asymptomatic hyperkalemia at early follow-up resolved over time, and overall potassium changes were not significantly different from controls. Given the high incidence of hyperkalemia, it was concluded that finerenone should be initiated at the lowest possible dose in transplant patients, with closer monitoring required compared to other patient populations.

There is no comprehensive study examining the effects of finerenone use on the serum levels of calcineurin inhibitors in organ transplant patients. Calcineurin inhibitors are drugs that significantly provide graft survival in organ transplant patients. However, they may have toxic effects at plasma levels outside the therapeutic range. At the same time, these drugs may cause calcineurin inhibitor-associated nephrotoxicity due to reducing renal blood flow, increasing oxidative stress, upregulating transforming growth factor-beta, and activating apoptotic genes [37,38,39]. It is thought that finerenone may have a positive effect on these pathological mechanisms. In an animal study on this subject, it was shown that finerenone added to standard treatment provided significant benefits in creatinine, interleukin-6, urinary neutrophil gelatinase-associated lipocalin, and blood pressure levels in a tacrolimus-mediated renal failure model compared to the group not receiving the drug [40]. In our study, no statistically significant difference was detected in the tacrolimus level at the 6-month follow-up. Long-term studies including histopathological evaluation are needed to examine the effects of finerenone on calcineurin-mediated nephropathy.

There are many etiological factors that can impair renal functions in organ transplant patients. The main ones are ischemia reperfusion injury, delayed graft function, rejection attacks, and calcineurin toxicity. It is known that MR is upregulated in many cases where renal damage occurs [12]. Based on this point, we think that the renoprotection shown in patients with CKD, whether diabetic or not, with the use of finerenone may also benefit organ transplant patients. However, considering the medications used in transplant recipients and patient-specific factors, aspects such as the pharmacokinetics of finerenone, its interactions with immunosuppressive agents, and the presence of various underlying causes of proteinuria require further elucidation. Although there is no published study on this subject, the ongoing EFFEKTOR (NCT06059664) study is an important study that will show the efficacy and safety of finerenone in kidney transplant recipients. Again, two studies (NCT02490904, NCT04450953) are ongoing to evaluate eplerenone in kidney transplant patients.

The most important limitation of our study is its retrospective design, which did not include randomization of subjects between the finerenone and control groups. Thus, there may have been differences between these groups accounting for the differences in proteinuria unrelated to taking finerenone. Additional limitations include the small number of cases, short follow-up period, retrospective data collection, lack of blood pressure monitoring, adjustment for confounding variables, and lack of biopsy data.

In conclusion, this study suggests that finerenone may contribute to the reduction of proteinuria in renal transplant patients as part of their therapeutic management and had no negative effect on GFR. Our study also identified a 15% dropout rate due to adverse events: 5% due to headache and flushing and 10% due to a risk of hyperkalemia, especially during the early phases of drug initiation, underscoring the importance of dose potassium surveillance. Prospective, randomized, controlled studies with large case series are needed on this subject.

## Figures and Tables

**Table 1:** Descriptive statics of clinical and biochemical parameters by groups.

Variables	Finerenon (*n* = 15) Median (Min–Max)	Control (*n* = 15) Median (Min–Max)	*p*
Age (years)	42 (24–61)	45 (19–59)	>0.05
Weight (kg)	75 (50–110)	70 (44.60–111)	>0.05
Height (cm)	170 (153–190)	171 (158–190)	>0.05
Transplant duration (months)	58 (13–150)	80 (12–142)	>0.05
BUN (mg/dL)	24 (14–49)	23(13–42)	>0.05
Creatinine (mg/dL)	1.85 (1.00–3.34)	1.52 (1.12–2.38)	>0.05
eGFR (mL/min/1.73 m^2^)	41.7 (25.80–81.17)	48.31(26.80–84.66)	>0.05
Proteinuria (mg/day)	2150 (600–5335)	1691 (403–7112)	>0.05
Uric acid (mg/dL)	6.9 (4.75–12.11)	6.7 (4.82–11.40)	>0.05
Potassium (mmol/L)	4.35 (3.29–5.00)	4.22 (3.30–5.00)	>0.05
ALT (U/L)	14 (5–36)	17 (8–33)	>0.05
LDL (mg/dL)	103 (38–257)	103 (40–215)	>0.05
TG (mg/dL)	147 (80–787)	101 (68–451)	>0.05
Tacrolimus (ng/mL)	7.70 (4.30–10.25)	6.52 (3.14–9.9)	>0.05
Donor Type (Living/Deceased)	11/4	7/8	>0.05
Sex (F/M)	5/10	9/6	>0.05
Smoking (+/−)	5/10	3/12	>0.05
Etiology			
HTN	9	12	
DM	1	0	
DM + HTN	1	0	
Others	4	1	
Unknown	0	2	

Notes: min: minimum value, max: maximum value. Abbreviations: eGFR: estimated glomerular filtration rate, F: female, M: male, ALT: alanine aminotransferase, BUN: blood urea nitrogen, HTN: hypertension, DM: diabetes mellitus, GN: glomerulonephritis, LDL: low-density lipoprotein, TG: triglyceride.

**Table 2:** Statistical distribution of baseline and 6th-month data in the finerenon and control groups.

Variables	GROUP 1 Median (Min–Max)			GROUP 2 Median (Min–Max)			*p* ^#^
	Baseline	6th Month	*p* *	Baseline	6th Month	*p* ^&^	
BUN (mg/dL)	24 (14–49)	26 (17–53)	>0.05	23 (13–42)	26 (18–72)	>0.05	>0.05
Creatinine (mg/dL)	1.85 (1–3.34)	1.82 (1–3.95)	>0.05	1.52 (1.12–2.38)	1.68 (0.91–4.26)	**<0.05**	>0.05
eGFR (mL/dk/1.73 m^2^)	41.7 (25.8–81.1)	40 (19.2–83.5)	>0.05	48.3 (26.80–84.66)	48.7 (17.00–69.35)	**<0.05**	>0.05
Proteinuria (mg/day)	2150 (600–5335)	1127 (80–3848)	**<0.05**	1691(403–7112)	1615 (420–5389)	>0.05	>0.05
Potassium (mmol/L)	4.35 (3.29–5)	4.32 (3.4–4.7)	>0.05	4.20 (3.3–5.0)	4.4 (3.4–5.5)	>0.05	>0.05
Tacrolimus (ng/mL)	7.7 (4.3–10.25)	6.28 (3.66–10.5)	>0.05	6.52 (3.14–9.9)	6.2 (4.7–10.2)	>0.05	>0.05
Uric acid (mg/dL)	6.9 (4.15–12.1)	6.27 (4.4–10.54)	>0.05	6.27 (4.82–11.4)	6 (4.96–10.8)	>0.05	>0.05
ALT (IU/L)	14 (5–36)	17 (5–58)	>0.05	13 (8–33)	16 (5–69)	>0.05	>0.05
LDL (mg/dL)	103 (38–257)	112 (36–191)	>0.05	103 (40–215)	115 (36–207)	>0.05	>0.05
TG (mg/dL)	147 (80–787)	173 (50–466)	>0.05	101 (70–502)	159 (37–581)	>0.05	>0.05

Notes: *: between baseline and 6th month in GROUP 1, ^&^: between baseline and 6th month in GROUP 2, ^#^: between groups, min: minimum value, max: maximum value. Abbreviations: BUN: blood urea nitrogen, eGFR: estimated glomerular filtration rate, ALT: alanine aminotransferase, LDL: low-density lipoprotein, TG: triglyceride.

## Data Availability

The data that support the findings of this study are available from the corresponding author upon reasonable request.

## Abbreviations

ACEIs	Angiotensin-converting Enzyme Inhibitors
ARBs	Angiotensin II Receptor Blockers
CKD	Chronic Kidney Disease
CKD-EPI	The Chronic Kidney Disease Epidemiology Collaboration
eGFR	Estimated Glomerular Filtration Rate
GFR	Glomerüler Filtration Rate
GLP-1	Glucagon-like Peptide-1
MRAs	Nonsteroidal Mineralocorticoid Receptor Antagonists
MR	Mineralocorticoid Receptor
SGLT2-Is	Sodium-glucose Co-transporter 2 Inhibitors
